# Probabilistic Detection of Indoor Events Using a Wireless Sensor Network-Based Mechanism

**DOI:** 10.3390/s23156918

**Published:** 2023-08-03

**Authors:** Lial Raja Al-Zabin, Ola A. Al-Wesabi, Hamed Al Hajri, Nibras Abdullah, Baidaa Hamza Khudayer, Hala Al Lawati

**Affiliations:** 1 Information Technology Department, Al-Zahra College for Women, Muscat 3365, Oman; hamad@zcw.edu.om (H.A.H.); hala_allawati@zcw.edu.om (H.A.L.); 2 Faculty of Computer Science and Engineering, Hodeidah University, Hodeidah P.O. Box 3114, Yemen; nibras@usm.my; 3 School of Computer Sciences, Universiti Sains Malaysia (USM), Gelugor 11800, Malaysia; 4 Information Technology Department, AlBuraimi University College, Al Buraimi 512, Oman; baidaa@buc.edu.om

**Keywords:** cluster head (CH), event detection, wireless sensor network (WSN)

## Abstract

Wireless sensor networks (WSNs) have been commonly utilized in event detection and environmental observation applications. The main aim of event detection is to define the presence or absence of an event. Various existing studies in the field of event detection depend on static or threshold values to reveal the occurrence of an event, which can result in imprecise sensor readings. Recently, many studies have utilized fuzzy logic to treat fluctuating sensor readings; as a result, they have decreased the number of false alarms created. However, there is some attention required when utilizing fuzzy logic. One aspect is that the efficiency and accuracy of the fuzzy membership function can be impacted by the utilization of heterogeneous sensors, which may increase the complexity of the fuzzy logic operation as the number of inputs rises. To address these issues, this paper proposes an approach named Probabilistic Collaborative Event Detection (PCED), which is a hybrid event detection technique that is based on a cluster WSN topology. The PCED approach utilizes a validated probabilistic technique for heterogeneous sensor nodes to transform sensing values into probability formulas and introduces a Cluster Head Decision Mechanism to make decisions based on the aggregated data from the sensors. The proposed approach employs fuzzy logic at the fusion center level to enhance the precision of event detection. The effectiveness of this method is thoroughly evaluated using MATLAB software, demonstrating an improvement in the probability of detection and a decrease in the probability of false alarms. PCED is compared to well-established event detection mechanisms such as the REFD mechanism. The results show that PCED reduces the occurrence of false alarms from 37 to 3 in certain scenarios, while improving detection accuracy by up to 19.4% over REDF and decreasing detection latency by up to 17.5%.

## 1. Introduction

Wireless sensor networks (WSNs) are proving to be highly beneficial for various applications that involve large-scale monitoring, especially in low-power and remote areas. As a result, they are increasingly being utilized for event-based detection mechanisms like fire and intrusion detection, both indoors and outdoors. However, the primary difficulty in designing a successful event detection mechanism using WSNs is to ensure that it is robust and reliable in order to reduce false alarms. The task of enhancing an effective event detection system utilizing WSNs that can improve accuracy and performance while reducing the possibility of false event detection is complicated, mainly in indoor scenarios. Various existing approaches struggle to achieve these goals [1].

Compared with the indoor environment, damaged sensors in outdoor environments are almost impossible to identify, maintain, or replace. An example of such a scenario is seen in cases in which WSNs are deployed in hilly areas. These sensors are usually thrown from a helicopter to monitor temperature or rainfall [2]. With their self-organization capability, sensors are organized into clusters after deployment, whereby cluster heads (CHs) collect and aggregate data and send it to the sink or base station (BS). Due to long-distance communication, most of the sensors will die when their battery dies, which completely disrupts the network. Therefore, extending the network’s lifetime can be achieved by clustering the network through a distributive mechanism [2,3].

Moreover, the detection strategies include collaboration detection and operation mode. The collaboration of the neighboring nodes in deciding whether sufficient evidence exists to trigger the propagation of an event detection report is of great benefit for energy conservation, accurate detection, and event processing delays [4]. To help firefighting operations, an alarm application based on Telos B motes [5] was proposed in [6]. A combination of temperature, light, and humidity sensors in harsh, hostile environments was used. The researchers considered a scattered WSN which consisted of several isolated WSNs. The scenario included sensor nodes destroyed by fire. The findings revealed that a system that achieves greater longevity by avoiding synchronization costs during idle periods can be successfully applied to fire breakouts when swift responses to such destructive events are needed. To recognize different classes of application-specific events, the authors of [7] proposed a system for the collaborative detection of an event in which instead of depending on a centralized BS for coordination or processing, sensing nodes collaborate to accurately conclude the occurrence of the event. Through the evaluation of the system using a WSN of 100 nodes deployed on the fence of a real-world construction site, it was found that the direct processing of the raw data on the nodes that only propagate events that have been detected contributes to a considerable reduction in communication overhead. Earlier, the authors of [8] proposed Data Service Middleware (DSWare), which can provide swift event detection services through collaborative correlations between different sensor observations according to real-time characteristics of events. This approach can distinguish between real occurrences of events and false alarms. Confidence functions were also used to define the relative importance of sub-events. Low detection rates trigger the instant report of the partial detections of these events. Therefore, a more accurate and comprehensive understanding of when and where events have happened can be achieved through collaborative detection. It does not contribute to a false-positive detection of the outcome. Nevertheless, exchanging collaboration information would delay the delivery of event reports; hence, the sink is not notified about the event swiftly, which affects the reliability and effectiveness of the applications.

Although the disk model is commonly utilized [9], it has drawbacks as the sensor node can only detect data within its radius range, causing events outside of the range to be missed [10]. Therefore, to overcome the limitations of the disk model, an attenuation model is utilized. This model considers the continuous attenuation of signals as sensors move further apart and the impact of noise on signal dispatch. In terms of data representation, researchers have reviewed the utilization of real values and a probabilistic approach. However, utilizing real values in sensing data presents two challenges. Firstly, the fusion center and cluster head must accommodate a range of sensor types which can be heterogeneous in nature. The second issue deals with fixed sensor thresholds. For instance, when a temperature sensor’s threshold is located at 55 °C and the genuine temperature for a fire is 45 °C, the sensor will only trigger an alarm when the temperature reaches 55 °C, resulting in a delay in detection and a risk of false alarms. To address this challenge, a probabilistic approach for sensing data representation has been devised which effectively removes the need for a predetermined threshold value [11]. Although a probabilistic technique has been developed to eliminate the reliance on fixed threshold values for sensor data representation, it has not been applied to different types of sensors that use different units and threshold levels. Therefore, this study utilized a probabilistic strategy to effectively represent data at the sensor level and address the variability in sensor types and units.

In addition, it is recommended that clustering-based WSNs are employed to improve the quality of sensing data before sending them to the fusion center to enhance decision making. To achieve this, recent techniques have combined data from multiple sensors to improve the accuracy of event detection [12]. However, the use of different types of sensors to detect an event and submitting their combined data to the cluster head can lead to an increase in the number of memberships, resulting in reduced detection accuracy and an increase in false alarms. Moreover, the majority of existing event detection methods depend on fuzzy logic techniques [13] to enhance decision-making accuracy, as fuzzy logic more closely resembles human cognition. For instance, instead of considering a fire event an incident with a temperature exceeding 55 °C and a smoke obscuration level above 15%, it can be perceived as an event with a high temperature and smoke. The utilization of fuzzy logic is much simpler and more straightforward in reality. To assess the credibility of each cluster and make a final decision regarding the presence of the target, fuzzy logic can be integrated [1,14]. 

While fuzzy logic is simpler to use, one major drawback is the exponential growth of rules with the increase in variables. This could lead to slow event detection and complexity as the number of rules in the rule base is m^n^ when there are n variables that can each take m values. In addition, the performance of fuzzy logic may be affected by the ranges and settings for membership function in heterogeneous sensors, leading to incorrect decisions as the number of inputs and evaluation levels increases.

The utilization of two types of sensors, namely, temperature and smoke, was explored in [14] to address the possibility of negative or zero readings in sensing values. However, this approach can also affect the decision-making process of fuzzy logic, resulting in an increased number of false alarms prior to the actual occurrence of an event. The paper’s subsequent sections are organized as follows: Section 2 presents an overview of related works. In Section 3, we present the problem definition and system formulation. The decision model and solution for event detection are detailed in Section 4. Section 5 features the simulation results. Lastly, we conclude this paper in Section 6.

## 2. Related Work

In this section, the focus is on the current body of research on WSNs and their application to event detection. This is a critical field of study that has garnered significant interest from researchers and scientists in recent times.

The application of the sensors’ disk model was initially deployed on a large scale. This technique is known as the disk model because the sensing field of the sensor resembles a disk shape. The sensor can detect a target if it is within the radius of the disk. The authors of [15] conducted a study on the search techniques and network detection capabilities of this model. They covered a range of sensor models and targets, such as mobile and stationary sensors and targets placed under random or optimal conditions. Additionally, the discussion included independent or globally coordinated search strategies and the use of stealthy or visible sensors. Throughout these various detection approaches, a Boolean sensing model with a fixed sensing radius was employed. In their research, the authors of [16] put forward a mathematical approach for analyzing the coverage of WSNs. The methodology involves calculating the number of active nodes needed to attain the desired coverage by comparing the range of a sensor node to that of the overall deployment area. If an event falls within a sensor’s range, it will be detected; otherwise, it will go undetected. The disk model has been deemed overly idealistic for a few reasons [10]. Due to the signal attenuation model, the signal detected by sensors experiences continual attenuation as the distance between them increases, and noise interference is also a factor. This means that the sensor measurements can be realistically simulated rather than providing a binary output. To enhance the study of Wireless Sensor Networks (WSNs) for event detection, researchers have turned to probabilistic event detection models. These models involve calculating and measuring probabilities associated with event occurrence, as well as related parameters, instead of relying on statistical computation and sample datasets.

In their study, the authors of [16] assessed the probability of detecting mobile targets in a scenario in which multiple sensors were randomly positioned to monitor a specific area of interest. The detection difficulty was related to the problem of line-set intersection and was dependent upon the perimeter constraints. The researchers found that the likelihood of detection was determined by the length of the sensor perimeters, with no influence from the shape of the perimeters. As a result, they developed a modified disk model that allows for the adjustment of the sensor range based on the perimeter constraints. 

Data fusion (DF) is a technique often utilized for enhancing the performance of detection systems [17]. DF involves the employment of a range of procedures to gather data from diverse sources, including sensors or CHs, to produce more precise inferences. Nowadays, there are two types of fusion centers in use: cluster-based and non-cluster-based fusion centers.

In [18], a hierarchical decision fusion technique was presented that aimed to improve the probability of network-wide detection. This method includes every sensor making its own local decision according to a signal attenuation model. After that, utilizing the closest neighboring sensors, a cluster-level sensor fusion is conducted to acquire new decision results. Lastly, sensors with positive fusion outcomes forward their data to the network fusion center, which makes the ultimate network-level decision. This fusion approach relies on certain assumptions, such as a higher-level decision yielding a comparable positive outcome or a negative one if the lower-level positive decision count exceeds a specific threshold. Despite these strategies’ potential advantages, they have encountered some challenges, such as the final decision always being based on more than half of the total number of positive local decisions made by the sensors. However, a smaller number of positive local decisions could still lead to a reliable final decision. To make the decision-making criteria more accurate, it is essential to consider more realistic indicators than just a single number. One fusion technique that accomplishes this is called “decision fusion” in which nodes send their local decision outcomes to a fusion center, where a final decision is made. It is important to note that another fusion technique exists called “value fusion” where nodes transmit raw energy data to the fusion center.

The final decision in a sensor network is typically based on measurements gathered from multiple nodes. However, the authors of [19] developed a wireless sensor network specifically designed for the early detection of mine fires. This system incorporates subsystems for data collection, data processing, and fusion monitoring. The research emphasized the network’s structure, scheduling mechanisms, and communication protocols, ensuring that they are suitable for this specific application. The authors of [20] introduced rapid algorithms for placing sensors in optimal positions based on a probabilistic data fusion model to enhance detection performance. In this approach, sensors transmit energy measurements to the *CH*, which compares the averages of all measurements against a present threshold. If the average is higher than the threshold, the *CH* concludes that a target is present; otherwise, it decides that no target is present. This research focused on the development of algorithms for sensor placement, ensuring maximum detection performance. The authors of [21] proposed a collaborative detection mechanism that relies on a unique data fusion approach to ensure the accurate deployment of sensors. They employed a “density first” clustering algorithm that organizes pre-selected surveillance locations into deployment units and places sensors to cover those locations. The algorithm uses a similar data fusion mechanism to make detection decisions. The focus of the research conducted in [22] was on the issue of barrier information coverage. The study proposed a solution that applied collaboration and information fusion between neighboring sensors to enhance intrusion detection while minimizing the number of active sensors needed to cover a barrier. To achieve this, a practical approach was suggested that only required a small number of active sensors to identify the coverage set. A virtual sensor was used to integrate the sensor readings, which led to a decision based on value fusion [23]. However, the value fusion approach has faced certain challenges, such as determining the threshold value. The majority of researchers who have used this method have not addressed these issues and have relied on an experiential value for the threshold. In [19], the majority rule was simplified to its most fundamental level where each sensor sends its individual decision to the CH, which then employs the majority rule to reach a final decision. Meanwhile, the previous fusion works have utilized a cluster-based network structure to enhance network performance and reduce communication expenses. 

In contrast, the authors of [14] suggested a fuzzy logic-based collaborative approach to robustly detect events. The researchers found that utilizing fuzzy values can enhance detection accuracy compared to the classification algorithms. However, the authors also noted that the limited memory of the nodes makes it challenging to store and distribute the growing number of rule bases. The authors of [1] reached a similar conclusion when they assessed their collaborative event detection mechanism utilizing clustered WSNs. The authors of [24] developed D-FLER, a distributed fuzzy inference engine, to detect fire events in residential settings using smoke and temperature sensors. By integrating individual sensor inputs with neighborhood observations via a distributed fuzzy logic engine, D-FLER was able to improve the accuracy of fire detection. The proposed sensor network technique used a temperature sensor and a maximum likelihood algorithm to fuse sensory information and detect the early signs of fire outbreaks in open areas such as forests and urban areas, as previously presented in [25].

The architecture of the proposed system comprises three subsystems, namely, sensing, computing, and localized alerting. The results of the study indicate that this system has been successfully implemented for early fire detection purposes. 

This paper proposes a method for event detection using sensors that employ a realistic attenuation signal model. The approach uses a probabilistic decision model for heterogeneous sensors to make more accurate decisions about event occurrence. The sensor data will be collected through clustering with the utilization of a weighting factor to facilitate decision making. Finally, a fuzzy logic method will be applied at the fusion center to produce a final decision.

## 3. Defining the Problem and Formulating the System

In this paper, an event detection system designed for indoor settings is proposed, with the purpose of environmental monitoring utilizing a variety of features such as gas, smoke, temperature, and light. In the PCED method, a group of heterogeneous sensors is utilized and spread uniformly all over the monitored domain to detect events such as fire blasts. By performing different types of measurements from their respective locations, these sensors generate a rich data stream that is aggregated and processed using a probabilistic model to provide accurate information. This information can be used to make informed decisions about safety measures, thus enabling swift and effective responses in emergency situations.

Let us consider a scenario in which the monitored region is partitioned into sub-areas, with each sub-area being monitored by a group of sensor nodes. Additionally, assume that the system administrator has organized these sensor groups into clusters.

**Figure 1:**
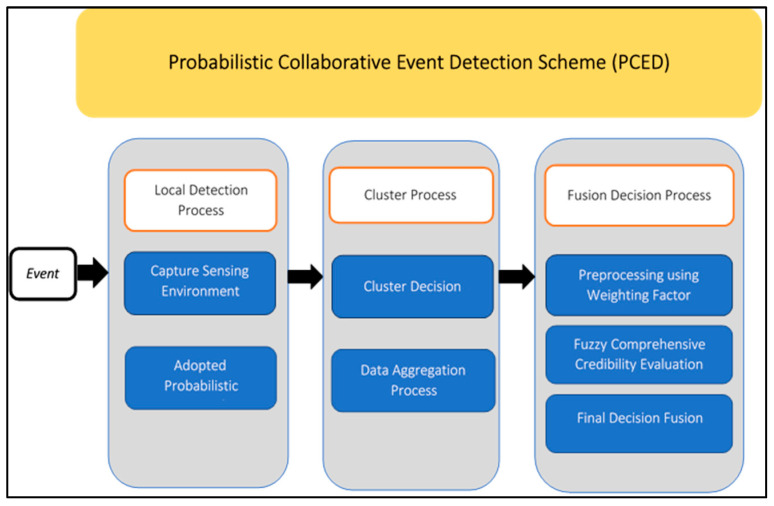
Stages of localized detection.

In this approach, the sensor nodes within a monitored region are organized into clusters, each consisting of different types of sensors and led by a cluster head (CH) possessing advanced computation and communication capabilities beyond that of a typical sensor node. Instead of transmitting data directly to the sink, the sensor nodes send local decisions to their respective CH, which is responsible for collecting measurements from its associated sensors, making cluster-level decisions, and aggregating data for fusion at the central data fusion point. The input data are further refined at the fusion center using a weighting factor to enhance the performance of the fuzzy logic and aid in making the final decision. The proposed event detection system (PCED) utilizes a hybrid approach that combines probabilistic and fuzzy logic methods to enhance detection performance in terms of both probability and accuracy. The PCED design is comprised of three stages, which are illustrated in Figure 1.

### 3.1. Local Detection Process

The local detection process depends on sensor detection, which can depend on any number of heterogenous sensors that will be integrated for local detection. PCED considers the case of fire, for which it proposes the use of four types of sensors (temperature, gas, light, and smoke sensors) to monitor and detect events based on adopting a probabilistic decision model. The sensors detect an event using a realistic attenuation signal model and can make a more reasonable decision about the presence of events based on the adoption of a probabilistic decision model as well. In the decision model, the measurement of values at each sensor is represented using the modified version of a probabilistic model developed in [11]. The event itself emits a signal, for example, a fire. A sensor can detect an event by measuring its energy. Meanwhile, the measurements obtained by sensors can also sometimes be contaminated by background noise [11]. In PCED, this observation is modeled as additive Gaussian noise. Assume that *H*_0_ represents the hypothesis that the target is absent, and *H*_1_ represents the other hypothesis that the target is present. In this paper, the methodology presented in Figure 1 is followed utilizing the following equations [11]. Thus, the energy measurement *e_i_* of sensor *i* is given by

(1a) $$H_{0}:e_{i}=e_{n}$$

(1b) $$H_{1}:e_{i}=e_{n}+e_{s}\left(d_{i}\right)$$

In this scenario, we have two hypotheses: *H*_0_, which assumes that the target is absent, and *H*_1_, which proposes that the target is present. Additionally, we define *e_n_* as the energy of the background noise, *e_i_* as the energy measurement obtained by sensor I, and *e_s_*(*d_i_*) as the energy of the attenuated signal at the position of sensor *i*.

The energy of the attenuated signal received by each sensor, *e_s_*(*d_i_*), can be computed as follows for each sensor:

(2) $$e_{s}\left(d_{i}\right)=\left\{\begin{array}{ll} \frac{s_{0}}{{{(d}_{i}/d_{0})}^{k}}, & d_{i}>d_{0}\;\\ s_{0}, & d_{i}\leq \;d_{0} \end{array}\right.$$

where *d*_0_ is a constant serving as the reference distance factor; *s*_0_ is the signal energy measured at a distance of *d*_0_; *k* is the attenuation factor, which typically ranges from 2 to 5; *d_i_* is the Euclidean distance between the target and sensor *i*; and *e_n_* approximately follows a Gaussian distribution, with a mean *μ* and a variance *σ*^2^.

Therefore, the total signal energy value of sensor *i* can be modeled as a Gaussian distribution, which is expressed as follows:

(3a) $${H_{0}:e}_{n}\sim N\left(\mu ,\sigma^{2}\right)$$

(3b) $${H_{1}:e}_{n}\sim N\left(\mu +e_{s}\left(d_{i}\right),\sigma^{2}\right)$$

**Figure 2:**
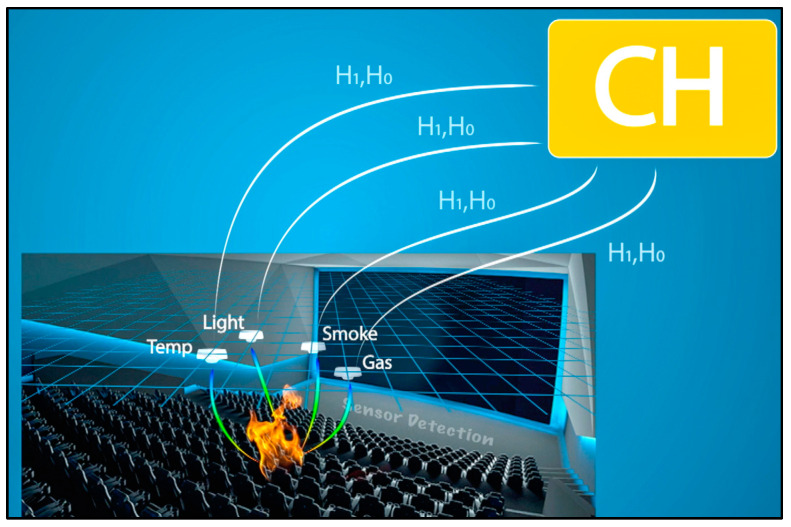
Sensor detection.

Consequently, each sensor generates a local decision based on a decision rule typically derived from the Likelihood Ratio Test [26]. This involves comparing the sensor’s signal energy measurement to a threshold *λ* determined by the measured value. If the measurement exceeds *λ*, the sensor returns a positive detection signal of 1, indicating that it has detected an event. If the measurement is below *λ*, the sensor returns a negative detection signal of 0, indicating that it has not detected an event. The probability of generating a positive detection signal when an event is truly present is known as the detection probability, while the probability of generating a false positive signal when an event is not present is referred to as the false alarm probability. Figure 2 is an overview of the sensor detection system. 

### 3.2. Process of Decision Making in Clusters

**Figure 3:**
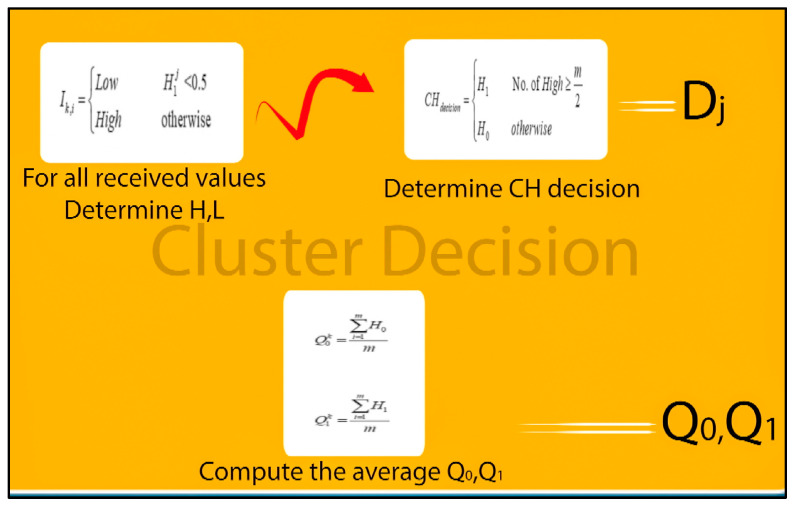
Cluster detection.

The *CH* of each sub-region makes the decision regarding the presence or absence of an event within that sub-region based on the local decisions communicated by the sensor nodes in the cluster, as shown in Figure 3. The binary decision from sensor node *i* in cluster j is denoted as $I_{j,i}$, where 1≤j≤k. If an event is detected, $I_{j,i}$ is set to 1; otherwise, it is set to 0. At the head of each cluster, the cluster decision is made based on the $N_{j}$ local decisions $I_{j,1},I_{j,2},\ldots ,I_{{j,N}_{j}}$ from $N_{j}$ local sensor nodes in the cluster. The variable $I_{j}$ indicates the number of 1 s among these local decisions:

(3c) $$I_{j}=\sum_{i=1}^{Nj}I_{j,i}$$

To arrive at a cluster decision at the CH, it is assumed that all local sensor nodes in cluster j have the same SNR and local detection probability, which is denoted as $p_{d_{j}}$. The prior probability is represented as $p=P[H=H_{1}]$. The optimal decision fusion rule utilized in this scenario is as follows:

(3d) $$H=\left\{\begin{array}{c} H_{1},\;\;\;\;\;\;\;\;\;\;\;\;\;\;\;\;IF\;\;\;\;\;\;\;\;\;\;\;\;\;\;\;\;\;I_{J}\geq \;{th}_{j}\\ H_{0},\;\;\;\;\;\;\;\;\;\;\;\;\;\;IF\;\;\;\;\;\;\;\;\;\;\;\;\;\;\;\;\;\;\;\;\;I_{j}<{th}_{j}\;\;\;\;\;\;\;\; \end{array}\right.$$

### 3.3. Fusion Center Process and Making the Final Decision

**Figure 4:**
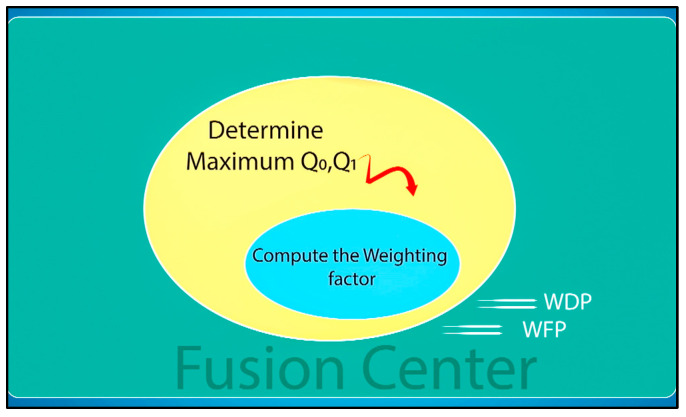
Pre-processing using weighting factors.

All decisions made by the CHs, along with their respective detection performances, are transmitted to the fusion center. The fusion center considers the credibility of each cluster to make the final decision, which is determined based on the distance from the event and the presence of concealed nodes and shadowing effects. Therefore, the final decision is made at the fusion center through the following three steps: 

Step 1: Pre-processing using a weighting factor, as shown in Figure 4. The weighting factor can give more confidence to a cluster that has a high detection probability and can result in minimal false alarms. In addition, the significance factor is used to eliminate some insignificant reading nodes by giving this cluster a lower weighting value, which may help to improve the overall performance and reduce the computation cost. 

Step 2: Fuzzy comprehensive credibility evaluation. Using fuzzy values (a set of values) instead of crisp ones (specific values) significantly improves the accuracy of event detection. Also, the fuzzy logic approach provides a higher degree of detection precision than other algorithms [9,14]. In this work, the credibility of a cluster is evaluated by a fuzzy comprehensive evaluator. This evaluator has two inputs: the probability of detection *Pd* and the probability of false alarm *Pf*. The output of the evaluator, *Cre*, is the credibility of the cluster that is being evaluated. The inputs and the output are characterized by the following term for the three fuzzy sets:


T(Pd)=T(Pf)=T(Cre)={Low, Medium, high}

**Figure 5:**
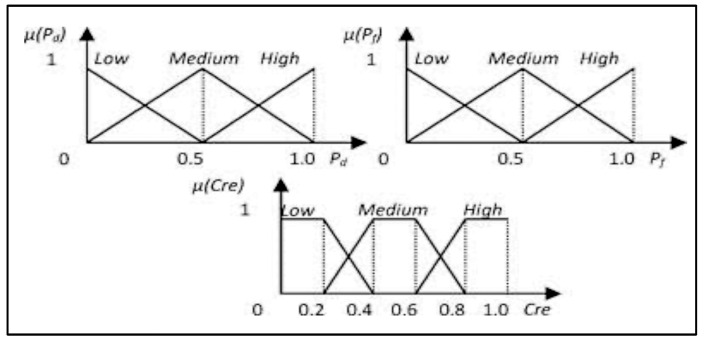
Membership functions of fuzzy sets [1].

The membership functions are illustrated in Figure 5.

The fuzzy inference rule set is proposed as follows:

- *IF* (*WPD is Low*) *THEN* (*Cre is Low*);
- *IF* (*WPD is Medium AND WPF is Low*) *THEN* (*Cre is Medium*);
- *IF* (*WPD is Medium AND WPF is Medium*) *THEN* (*Cre is Medium*);
- *IF* (*WPF is High*) *THEN* (*Cre is Low*);
- *IF* (*WPD is High AND WPF is Low*) *THEN* (*Cre is High*);
- *IF* (*WPD is High AND WPF is Medium*) *THEN* (*Cre is Medium*).

**Figure 6:**
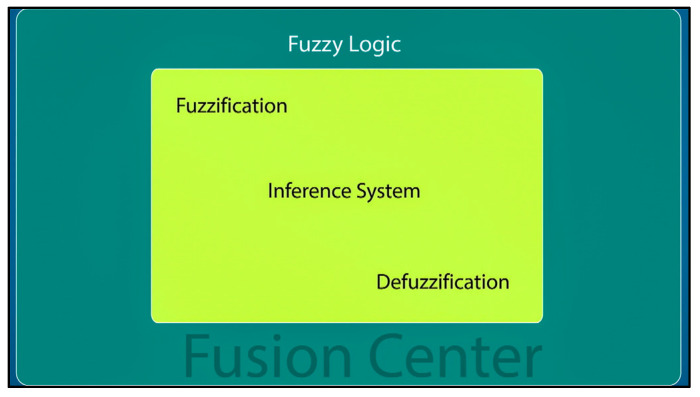
Fusion center-based fuzzy logic.

The defuzzification procedure used in this evaluator is the center of area method. The output of the defuzzification procedure is the credibility of the evaluated cluster, as shown in Figure 6.

Step 3. Final decision fusion. The final decision will be made based on the cluster decisions and their corresponding weight values. The decision from a cluster with a higher credibility receives a greater weight and vice versa. From this aspect, the weight *w_j_* of the *j*-*th* cluster is obtained by normalizing the credibility as follows:

(3e) $$w_{j}=\frac{{Cre}_{j}}{{Cre}_{max}}$$

**Figure 7:**
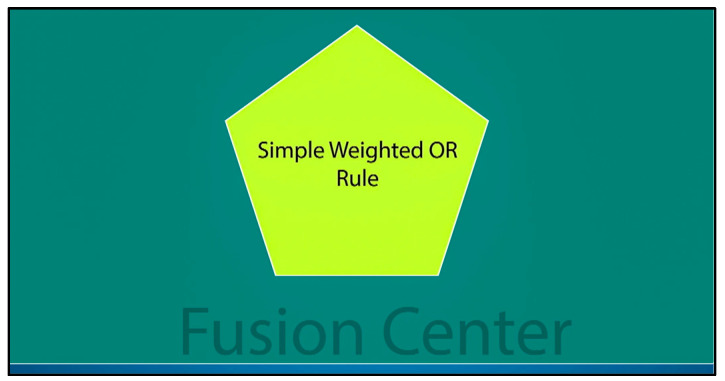
Final decision.

In conclusion, the final decision is made based on the simple weighted OR rule, as shown in Figure 7.

## 4. PCED Mechanism: Proposed Design

This study utilizes the advantages of existing related works by using a hyper probabilistic approach to convert the measured value at the sensors into a probability instead of directly using the value for the final decision in the fusion center. The *CH* plays a key role in gathering the measured values from sensors that are in the cluster to perform cluster-level decisions and aggregate the data to be sent to the fusion center. At the fusion center, the input data are improved by proposing weighting factors based on the performance of fuzzy logic. In terms of network topology, a cluster-based WSN structure was considered in this study in which the monitored area is divided into sub-areas and groups of sensor nodes monitor these sub-areas. The designed approach suggests adopting the probabilistic approach and modifying it to be suitable for event detection. Heterogeneous sensors are used in this research to achieve the designed objective which require different design requirements such as mean, variance, and threshold values. Using different types of sensors can help the detection mechanism in terms of detection accuracy and the confidence in an event being present or absent.

### 4.1. Probabilistic Model for Local Detection Design 

Following the design methodology described in Section 3, due to the advantages of using probabilistic models in event detection, this subsection describes the probabilistic model in a heterogeneous WSN in which different types of sensors can be used to improve the event detection mechanism in terms of accuracy and detection efficiency. The use of different types of sensors is inspired from the hypothesis that using heterogeneous sensors in many event detection mechanisms is advantageous because more than one type of sensor can detect the same event and thus corroborate it. For example, a fire event usually occurs with a combination of a high temperature, i.e., detected by temperature sensors, a high level of smoke, i.e., detected by smoke sensors, and a high light intensity, i.e., detected by light sensors. This study proposes that all different sensor modalities should be located close to each other so that they are approximately the same distance *d* from the event. Indeed, sensors perform detection by measuring energy emitted from the source of an event, and this energy attenuates with distance from that source. Thus, the signal energy received by sensor *i* at a distance *d_i_* from the source of an event is computed using Equation (4a) [11]:

(4a) $$e_{i}^{j}\left(d_{i}\right)=S_{0}+w^{j}(d_{i})$$

where $S_{0}$ is the energy measured at a distance *d*_0_, and $w^{j}(d_{i})$ is the attenuation factor of the emitted energy from source of the event with a distance di for sensors of type *j*. The formula of $w^{j}(d_{i})$ mainly depends on the propagation behavior. The PCED mechanism is mainly proposed for indoor environments and, to the best of our knowledge, Equation (4a) has been used in several existing studies in the field of event detection in indoor environments; thus, Equation (4a) should use the same attenuation factor values for all types of sensors, and the received energy as the signal attenuation is a function of distance. In addition, further study should be conducted to study the behaviors of different sensing modalities. Sensor measurements in a normal situation (the absence of an event) are contaminated by thermal noise (additive random noise) from the environment and the sensor itself. Thus, for each sensor of type *j*, based on the hypothesis of the absence of an event $H_{0}^{j}$ or the hypothesis of the presence of an event $H_{1}^{j}$ and according to Section 3.1, the energy at a sensor follows a Gaussian distribution, with a mean value equal to µ and a variance equal to *σ*^2^. Therefore, for different types of sensor nodes used in the monitoring area, the total measured value of the sensing signal at sensor *i* for sensing type *j* can be represented by adopting Equations (4b) and (4c) as follows:

(4b) $$H_{0}^{j}:\;e_{i}^{j}\sim N(\;\mu ,\;\sigma^{2})$$

(4c) $$H_{1}^{j}:\;e_{i}^{j}\sim N(\;e_{s}^{j}+\mu ,\;\sigma^{2})$$

In this paper, ($H_{0}^{j}$) indicates the values of a situation in which an event is absent, and it also represents a normal range of values such the temperature for an indoor room. Equations (4b) and (4c) can be applied in the probability density function (PDF) formula of a Gaussian distribution. Thus, the PDF of the sensing measurements at sensor $e_{i}^{j}$ can be expressed as follows:

(4d) $$H_{0}^{j}:\;P_{f}\left({\;e}_{i}^{j}\;\right)=\frac{1}{\sqrt{2\pi \sigma \;}}e^{\frac{\left(\;{e\;}_{i}^{j}-\mu \;\right)}{2\sigma^{2}}}$$

(4e) $$H_{1}^{j}:\;P_{f}\left({\;e}_{i}^{j}\;\right)=\frac{1}{\sqrt{2\pi \sigma \;}}e^{\frac{(\;{e\;}_{i}^{j}-\mu -{e\;}_{i}^{j}\left(d\right)\;{)}^{2}}{2\sigma^{2}}}$$

In traditional event detection, sensors compare their signal energies to a fixed threshold *λ* to determine the presence or absence of an event. If the signal energy exceeds *λ*, the sensor outputs a value of 1 to indicate the presence of an event; otherwise, it outputs 0 to indicate the absence of an event. However, to make event detection more accurate and realistic, probabilistic techniques are used. With this technique, sensor nodes convert their detection results into probabilities that indicate the likelihood of an event being present or absent. These probabilities are then sent to the corresponding *CH*. To accomplish this, two probability distributions ($H_{0},H_{1},{(P}_{f},P_{d}$)) are employed. The likelihood of making a positive determination regarding the existence of an event in the absence of one is referred to as the false alarm probability. On the other hand, a true alarm (detection probability) is the probability of making a positive determination when an event is present.

We can express the probabilities of false alarm detection and true detection for a sensor *i*, denoted by $P_{F}^{i,\;j}$ and $P_{D}^{i,\;j}$, respectively, for a sensing type *j*, by utilizing Equations (4d) and (4e) as follows:

(4f) $$P_{F}^{i,\;j}=P_{r}\left(e_{i}^{j}\geq \lambda_{j\;}|H_{0}\right)=\int_{\lambda_{j}}^{\infty }P_{f}\left({\;e}_{i}^{j}\;\right){de}_{i}^{j}$$

(4g) $$P_{D}^{i,\;j}=P_{r}\left(e_{i}^{j}\geq \lambda_{j\;}|H_{1}\right)=\int_{\lambda_{j}}^{\infty }P_{d}\left({\;e}_{i}^{j}\;\right){de}_{i}^{j}$$

Equations (4f) and (4g) indicate that a measurement value closer to the event for a sensor type *j* can lead to a higher probability of detection and a lower likelihood of a false alarm. In these equations, the symbol $\lambda_{j\;}$ represents the threshold value for a sensor type *j*, while $P_{r}$ (·) denotes the probability formula.

Through the substitution of Equations (4d) and (4e) into Equations (4f) and (4g), respectively, we obtain the following:

(4h) $$P_{F}^{i,\;j}=P_{r}\left(e_{i}^{j}\geq \lambda_{j\;}|H_{0}\right)=\int_{\lambda_{j}}^{\infty }\frac{1}{\sqrt{2\pi \sigma \;}}\;e^{-\frac{{\left(\;{e\;}_{i}^{j}-\mu \;\right)}^{2}}{2\sigma^{2}}}{de}_{i}^{j}$$

(4i) $$P_{D}^{i,\;j}=P_{r}\left(e_{i}^{j}\geq \lambda_{j\;}|H_{1}\right)=\int_{\lambda_{j}}^{\infty }\frac{1}{\sqrt{2\pi \sigma \;}}\;e^{-\frac{{\left(\;{e\;}_{i}^{j}-\mu -{e\;}_{i}^{j}\left(d\right)\right)}^{2}}{2\sigma^{2}}}{de}_{i}^{j}$$

Each sensor transmits this information to its corresponding cluster head to facilitate the subsequent decision-making process.

**Figure 8:**
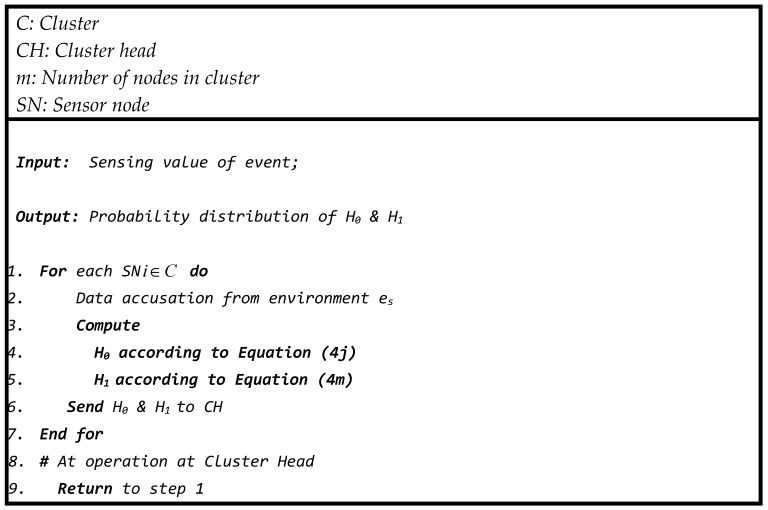
Pseudo-code of procedures in sensor node.

The following pseudo-code (Figure 8) is performed at every sensor node in the network according to the operational description of the probabilistic approach to convert the sensing value into a probability. Each sensor sends this information to its corresponding cluster head for further decision process.

### 4.2. Cluster Head Decision Making and Data Aggregation

The *CH* receives the computed detection probability ($P_{D}^{i,\;j}$) and false alarm probability ($P_{F}^{i,\;j}$) for each sensor from all its member sensor nodes. Most of these probabilities received from the sensor nodes within cluster *k* are used to make a decision at the *k*th cluster head. The node deployment is uniformly distributed across all clusters so that each cluster head node receives m detection probability values (*I*_*k*,1_, *I*_*k*,2_, …, *I*_*k*,*m*_), where m represents the number of sensor nodes participating in the cluster. At the cluster heads, the received probabilities of *H*_0_ and *H*_1_ can be expressed as follows:

(4j) $$H_{0}^{k}=\left\{\begin{array}{c} H_{0}^{1}\;:\;P_{F}^{i,\;1}=\int_{\lambda_{1}}^{\infty }\frac{1}{\sqrt{2\pi \sigma^{2}}}\;e^{-\frac{{\left(\;{e\;}_{i}^{1}-\mu \;\right)}^{2}}{2\sigma^{2}}}{de}_{i}^{1}\;\\ H_{0}^{2}\;:\;P_{F}^{i,\;2}=\int_{\lambda_{2}}^{\infty }\frac{1}{\sqrt{2\pi \sigma^{2}\;}}\;e^{-\frac{{\left(\;{e\;}_{i}^{2}-\mu \;\right)}^{2}}{2\sigma^{2}}}{de}_{i}^{2}\\ .\\ .\\ H_{0}^{m}\;:\;P_{F}^{i,\;m}=\int_{\lambda_{m}}^{\infty }\frac{1}{\sqrt{2\pi \sigma^{2}\;}}\;e^{-\frac{{\left(\;{e\;}_{i}^{m}-\mu \;\right)}^{2}}{2\sigma^{2}}}{de}_{i}^{m} \end{array}\right.$$

(4k) $$H_{1}^{k}=\left\{\begin{array}{c} H_{1}^{1}\;:\;P_{F}^{i,\;1}=\int_{\lambda_{1}}^{\infty }\frac{1}{\sqrt{2\pi \sigma^{2}}}\;e^{-\frac{{\left(\;{e\;}_{i}^{1}-\mu -{e\;}_{i}^{1}\left(d\right)\;\right)}^{2}}{2\sigma^{2}}}{de}_{i}^{1}\;\\ H_{1}^{2}\;:\;P_{F}^{i,\;2}=\int_{\lambda_{2}}^{\infty }\frac{1}{\sqrt{2\pi \sigma^{2}\;}}\;e^{-\frac{{\left(\;{e\;}_{i}^{2}-\mu -{e\;}_{i}^{2}\left(d\right)\;\right)}^{2}}{2\sigma^{2}}}{de}_{i}^{2}\\ .\\ .\\ H_{1}^{m}\;:\;P_{F}^{i,\;m}=\int_{\lambda_{m}}^{\infty }\frac{1}{\sqrt{2\pi \sigma^{2}\;}}\;e^{-\frac{{\left(\;{e\;}_{i}^{m}-\mu -e_{i}^{m}\left(d\right)\;\right)}^{2}}{2\sigma^{2}}}{de}_{i}^{m} \end{array}\right.$$

**Figure 9:**
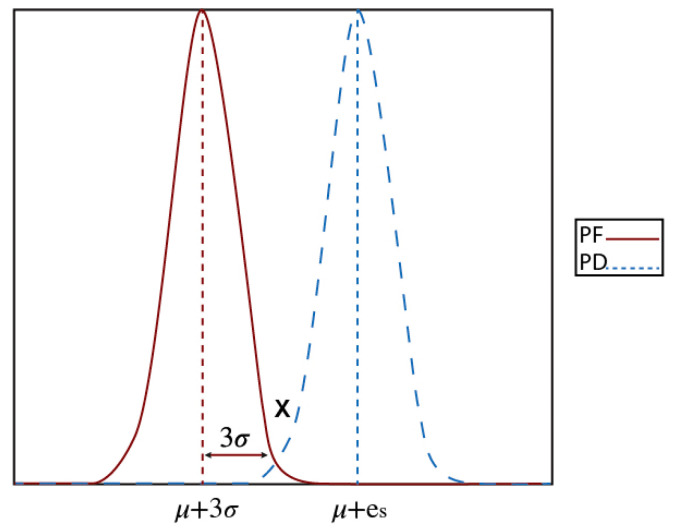
Probability density function for detection and false alarm.

In reference [10], a theorem is presented that states that if the attenuated signal energy (*d_i_*) from the target received by a sensor *i* is greater than six times the noise variance *σ*, then its local false alarm probability will be no greater than approximately 0.1%, and its local detection probability will be no less than approximately 99.9%. This theorem can be quite useful in determining the relationship between the two probability density functions (PDFs) of *H*_0_ and *H*_1_ for single-sensor detection, as illustrated in Figure 9. In this figure, x represents the intersection point between the two PDF curves, and *e_i_*(*x*) is the measured value at *x*. As a general trend, the probability of a false alarm decreases as *e_i_* increases until it reaches *x* (which is approximately 0.1%). Conversely, the detection probability increases proportionally to *e_i_* for all values greater than *x*. If any of the *e_i_* values are less than *x*, then an event is considered absent, and the decision of event (1) is considered a false alarm.

Based on the characteristics of the Gaussian distribution, if the false alarm probability is expressed as the probability of *e_i_* being greater than *x*, then it would be less than approximately 0.1% if *x* is larger than *µ* + 3*σ* [9] for Pf(*e_i_*(*d*) > *e_i_*(*x*)). Here, *µ* and *σ* represent the mean and the standard deviation of the noise, respectively. Conversely, if an event is present, Pd(*e_i_*(*d*) > *e_i_*(*x*)) can be greater than approximately 99.9% under the same condition of *x*.

Based on the correlation between the PDFs, the connection between *x* and *d_i_* can be expressed as follows:

(4l) $$\begin{array}{l} \mu +e_{s}\left(d_{i}\right)-\mu =2\left(e_{x}-\mu \right)\\ x=\frac{e_{x}}{2}+\mu \end{array}$$

To ensure that the probability of false alarms is kept below 0.1% at position *x*, the value of *e_i_*(*x*) needs to be adjusted to be higher than *µ* + 3*σ*.

$$\begin{array}{l} e_{x}\geq \mu +3\sigma ,\\ e_{x}\geq 6\sigma \end{array}$$

It should be emphasized that in an event detection system, an increase in the measured value at the sensor leads to a higher probability of detecting an event. Consequently, the probability of detection *P_D_(e_i_)* at position x is roughly equivalent to the probability of a false alarm *P_F_(e_i_).*

$$P_{F}\left(e_{x}\right)=P_{D}\left(e_{x}\right)$$

Nevertheless, the condition mentioned above is not evident in Equation (4l), which indicates that *P_D_(e_x_)* exceeds 99.9% for all values of *e_i_* less than *e_x_* but declines for values greater than *e_x_*. Consequently, it is necessary to revise Equation (4l) to comply with the design specifications that require the detection probability to increase with the sensing value. As a result, the equation for *H*_1_ should be written as follows:

(4m) $$H_{1}^{k}=\left\{\begin{array}{c} H_{1}^{1}\;:\;P_{F}^{i,\;1}=1-\int_{\lambda_{1}}^{\infty }\frac{1}{\sqrt{2\pi \sigma^{2}}}\;e^{-\frac{{\left(\;{e\;}_{i}^{1}-\mu -{e\;}_{i}^{1}\left(d\right)\;\right)}^{2}}{2\sigma^{2}}}{de}_{i}^{1}\;\\ H_{1}^{2}\;:\;P_{F}^{i,\;2}=1-\int_{\lambda_{2}}^{\infty }\frac{1}{\sqrt{2\pi \sigma^{2}\;}}\;e^{-\frac{{\left(\;{e\;}_{i}^{2}-\mu -{e\;}_{i}^{2}\left(d\right)\;\right)}^{2}}{2\sigma^{2}}}{de}_{i}^{2}\\ .\\ .\\ H_{1}^{m}\;:\;P_{F}^{i,\;m}=1-\int_{\lambda_{m}}^{\infty }\frac{1}{\sqrt{2\pi \sigma^{2}\;}}\;e^{-\frac{{\left(\;{e\;}_{i}^{m}-\mu -e_{i}^{m}\left(d\right)\;\right)}^{2}}{2\sigma^{2}}}{de}_{i}^{m} \end{array}\right.$$

**Figure 10:**
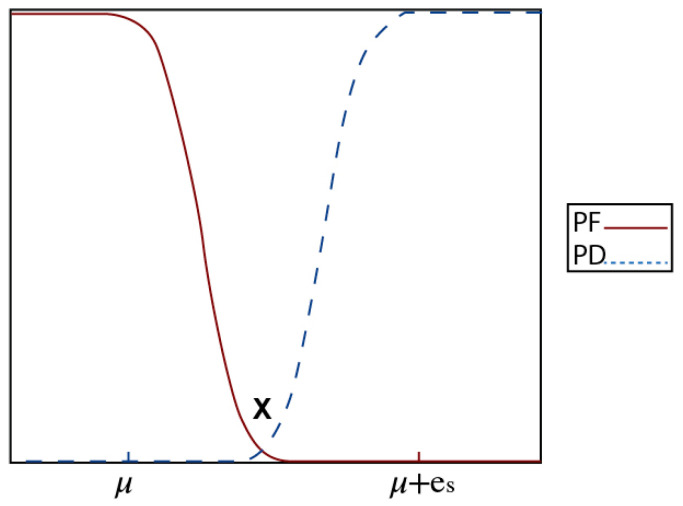
Analysis of probability distributions and false alarm detection.

Consequently, Figure 10 portrays the false alarm and detection probabilities at the sensor level, which are derived from Equations (4j) and (4m).

The decision-making process at cluster *CH* employs probability information from the sensor readings to arrive at accurate decisions. To design the decision-making mechanism at *CH*^k^, a rule-based approach was utilized. Typically, the likelihood of an event occurrence is strengthened when multiple nodes report elevated readings from the same sub-area. As a result, this supports the hypothesis that various sensors detecting high values indicate the presence of an event. This study proposed a rule-based approach for event detection at *CH*s.

Indeed, increasing the number of granularity levels (such as very low, low, …, high, and very high) may potentially enhance the accuracy of a system. However, in a resource-constrained environment like a WSN, the designer of an event detection system must reduce the number of membership sets to maintain accuracy while minimizing the complexity of rules and memory usage. To implement these rules, the probability values received from the sensors are translated into two levels: high and low. The range of values (0–1) in the distribution is split into two levels using the following approach:

(4n) $$I_{k,i}=\left\{\begin{array}{c} Low\;\;\;\;\;\;\;\;\;\;\;H_{1\;\;}^{j}<0.5\\ High\;\;\;\;\;\;\;\;\;\;\mathrm{otherwise} \end{array}\right.$$

As each cluster employs distinct types of sensors, the decision at a *CH* can be based on the majority rule. If m denotes the number of sensors in each cluster, a *CH* requires at least half (*m*/2) of the sensors reporting high values to confidently confirm the occurrence of an event. This can be expressed as follows:

(4o) $${CH}_{decision}=\left\{\begin{array}{cc} H_{1} & \mathrm{No}.\;\mathrm{of}\;\mathrm{High}\geq \frac{m}{2}\\ H_{0} & \mathrm{otherwise} \end{array}\right.$$

where *CH* decision refers to the decision made at the *CH*.

If *A* indicates the presence of an event and $B_{i}^{j}$ represents the present measurement value obtained by a sensor *i*, the value is computed for each value in the r-level set that includes N different values for both *H*_0_ and *H*_1_. Here, *i* = 1, 2, …, *m*, as there are m sensors in the cluster. Assuming an event is absent under the hypothesis *H*_0_, the estimated probability *Q*_0_ represents the likelihood of the cluster receiving readings from its m sensors (false alarm). Assuming the hypothesis *H*_1_ is true and an event is present, *Q*_1_ denotes the cluster probability of sensors collecting their own data. The aim of this method is to determine a shared detection probability and false alarm probability for all sensors in the cluster. Hence, the detection probability and false probability for a cluster *k* can be determined through the following computation:

(4p) $$Q_{0}^{K}=\frac{\sum_{i=1}^{m}H_{0}}{m}$$

(4q) $$Q_{1}^{K}=\frac{\sum_{i=1}^{m}H_{1}}{m}$$

**Figure 11:**
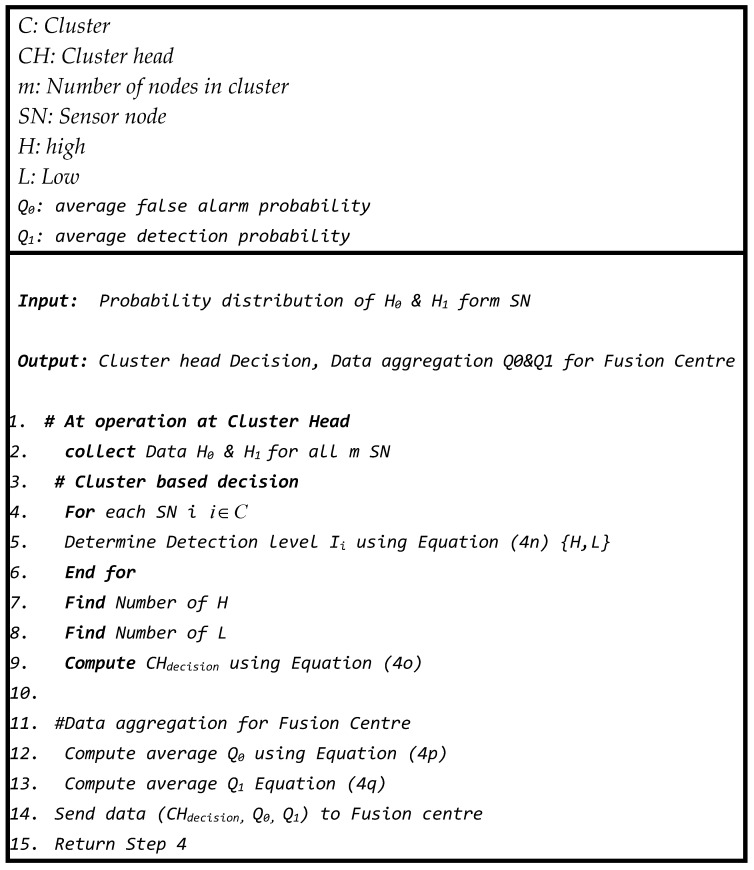
Pseudo-code of procedures at cluster head.

The following pseudo-code (Figure 11) is executed at every *CH* node in the network according to the operational description of the probabilistic approach of the sensor node to convert the measured value into a probability.

The CHs send their *Q*_0_ and *Q*_1_ estimates to the fusion center for further detection and final decision making. The data from the clusters are gathered at the fusion center and fuzzy logic are employed to make the final decision. However, it should be noted that different clusters may have varying data values depending on their distance from the event source. As such, this factor should be considered during the development of the fusion center and in the final decision-making process.

### 4.3. Pre-Processing and Aggregation of Data

The distance from the source of the event greatly affects the decisions made by the sensor nodes and their corresponding CHs such that clusters located closer to the source of the event have higher detection probabilities with a greater confidence and weight. To account for this, this study proposed the use of a weighting factor for each cluster that reflects the significance of its data readings in the final event detection decision. The weighting factor can be obtained through the following formula.

Identify the detection probability with the highest value:

(4r) $$Q_{1,max}=\arg\;\max\;(Q_{1}^{1},\;Q_{1}^{2},\ldots Q_{1}^{k}..Q_{1}^{NC})$$

where *NC* represents the total count of clusters.

The weighting factor for each cluster can be calculated based on the mean distance vector for a cluster *k* using the following formula:

(4s) $${∆}_{1}^{k}=Q_{1,max}-Q_{1}^{k}$$

The weighting factor can be expressed as follows:


(4t) $$W_{1}^{k}=\frac{1-{∆}_{1}^{k}}{Q_{max}}$$

By examining the equation above, we can see that the highest weight that can be allocated to a cluster is determined as follows:

(4u) $$W_{1,max}=\frac{1}{Q_{1,max}}$$

The minimum weight can be determined in cases in which the sensor nodes in a cluster are far from the event and cannot measure any data, resulting in an approximate detection probability $Q_{1}^{k}$ of 0. This can be expressed as follows:

(4v) $$W_{1,min}=\frac{1-Q_{1,max}}{Q_{1,max}}$$

Hence, the range of weighting factors $W_{1}^{k}$ can be defined as follows:


(4w) $$\;\;\;\;\frac{1-Q_{1,max}}{Q_{1,max}}\;\leq W_{1}^{k}\leq \;\frac{1}{Q_{1,max}}$$

The same procedure can be applied to obtain the weighting factor for the false alarm probability, which can be expressed as follows:

(4x) $$\;\;\;W_{0}^{k}=\frac{1-Q_{0,max\;}+Q_{0}^{k}}{Q_{0,max}}$$

The range of the weighting factor $W_{0}^{k}$ for the false alarm probability can be expressed as follows:

(4y) $$\;\;\;\frac{1-Q_{0,max}}{Q_{0,max}}\;\leq W_{0}^{k}\leq \;\frac{1}{Q_{0,max}}$$

The utilization of weighting factors $W_{0}^{k}$ and $W_{1}^{k}$ can enhance the effectiveness of fuzzy logic by limiting the number of inputs to just two, which in turn reduces the complexity, particularly during the fuzzification process while applying the membership function and set of fuzzy rules. The proposed modification can potentially improve the accuracy of event detection by considering the decision made by each cluster, which is weighted by the assigned factor, in combination with the decisions from all other clusters in the monitored area.

The fusion center receives inputs consisting of the detection probability $Q_{1}^{k}$ and false alarm probability $Q_{0}^{k}$, along with the *CH* decision. These inputs are then processed to obtain the weighted detection probability (WDP) and weighted false alarm probability (WFP). The equations for calculating WDP and WFP are as follows:

(4z) $$WDP=\left\{\begin{array}{c} 1\;{\;\;\;\;\;\;\;\;\;\;\;\;\;\;\;\;CH}_{decision}=H_{1}\\ {\;\;\;\;W}_{1}^{k}.\;\;Q_{1}^{k}\;\;\;\;\;\;otherwise\;\;\;\;\;\;\;\;\;\;\;\;\;\;\; \end{array}\right.$$

(4A) $$WFP=\left\{\begin{array}{c} 1\;{\;\;\;\;\;\;\;\;\;\;\;\;\;\;\;\;CH}_{decision}=H_{0}\\ {\;\;\;\;W}_{0}^{k}.\;\;Q_{0}^{k}\;\;\;\;\;\;otherwise\;\;\;\;\;\;\;\;\;\;\;\;\;\;\; \end{array}\right.$$

The weighting factor can provide a greater weight to the cluster with a higher detection probability, ultimately leading to fewer false alarms.

### 4.4. Process of Applying Fuzzy Logic

**Figure 12:**
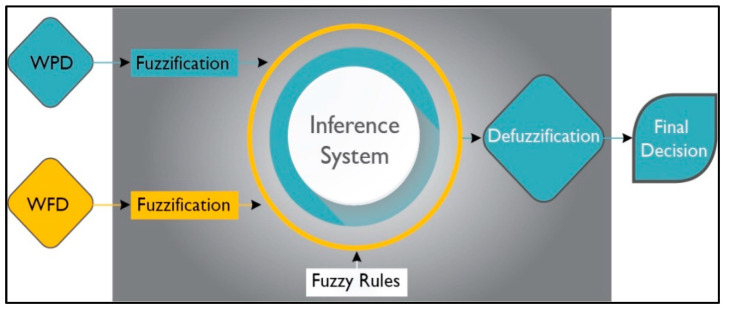
Fuzzy logic system (FLS).

The selected fuzzy logic system (FLS) for the proposed event detection mechanism is illustrated in Figure 12. The FLS uses the WDP and WFP as inputs. Each fuzzy cluster is evaluated using fuzzy logic, and the final decision is based on the results from all fuzzy clusters. A set of predetermined rules for event detection is created using if–then expressions, which are then used to process the fuzzified values. The inference mechanism is responsible for mapping input fuzzy sets to output fuzzy sets based on a predefined rule.

In the end, the DE fuzzifier computes the crisp output value as the final result. These crisp output values indicate the cluster’s detection results as well as the corresponding control action that needs to be taken.

#### 4.4.1. Fuzzification 

**Figure 13:**
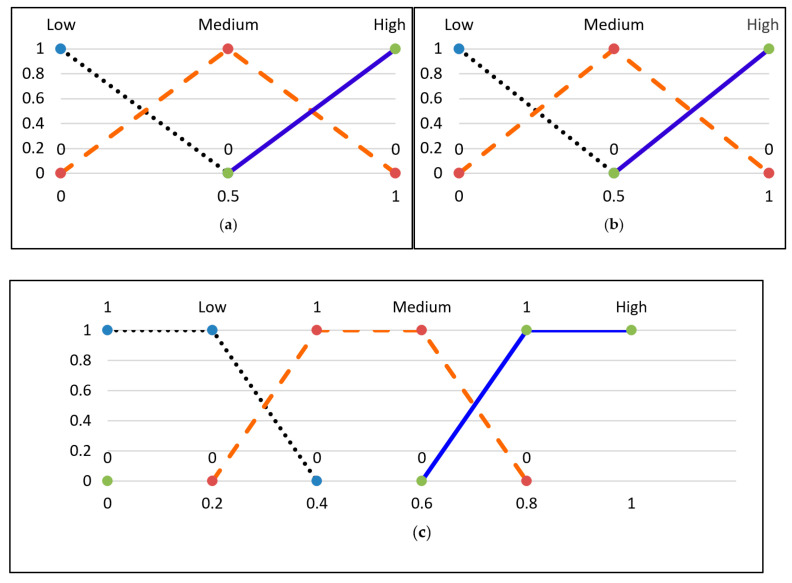
Fuzzy set membership functions and credibility. (**a**) Weighted detection probability; (**b**) weighted false alarm probability; (**c**) credibility.

The membership functions were created with triangular shapes to determine the certainty of how a crisp value relates to a specific linguistic term. These linguistic terms are represented in fuzzy logic, and Figure 13 shows the membership function for weighted detection and weighted false alarm probability. Each crisp input value is transformed into three levels, such as Low (0.0), Medium (0.8), and High (0.2), based on the detection probability membership function. For instance, if the weighted detection probability is 0.6, it will be transformed accordingly. One should give more weight to a cluster that has a higher chance of detection and a lower probability of false alarms. Therefore, to evaluate the credibility of each cluster, a fuzzy evaluator takes two inputs representing the probability of detection and false alarms (WDP and WFP) and produces an output representing the credibility (Cre) of the cluster under consideration.

#### 4.4.2. Fuzzy Inference System

The Fuzzy Inference System (FIS) maps a fuzzy set of two inputs, WDP and WFP, to an output representing credibility. The Mamdani method is widely used in FIS, and it is the chosen technique for the proposed research. The subsequent paragraphs explain the process of designing the FIS for detecting the proposed event. The input values fall between 0 and 1 and indicate the extent to which they belong to the appropriate fuzzy sets, as illustrated in Figure 13a,b. Subsequently, a set of linguistic statements is established as a set of rules to translate the fuzzy input set into credibility levels, which are also expressed using linguistic terms such as Low, Medium, and High. Table 1 shows all the potential rules for the devised fuzzy logic.

As the number of linguistic variables and inputs increases, the size of the rule base set grows exponentially, making it essential to minimize the number of rules in the fuzzy rule base during the design process to prevent complex scenarios and memory limitations. Consequently, Table 1 can be simplified and updated to Table 2.

**Figure 14:**
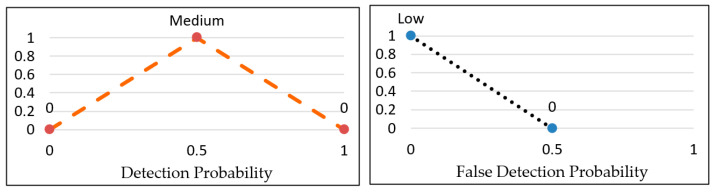
Application of rule 2 on Table 2 as an example.

Subsequently, the OR method (maximum) is employed as the fuzzy operator to compute the credibility level (tipping calculations) for the rules. For instance, if WDP = 0.4 and WFP = 0.6, rule 2 is utilized, and the fuzzy membership values of the rule are Medium (0.6) for WDP and Low (0.2) for WFP. As a result, the fuzzy operator selects the maximum value from the two membership values, which is 0.6, as demonstrated in Figure 14.

The output of each rule needs to be linked with a value, which is achieved by applying the implication method to the results from the fuzzy operator (as in the previous example where the output was 0.6). Once the implication procedure is completed for each rule, an aggregation process is carried out to enable efficient decision making, as the decision is based on all the tested rules. The maximum aggregation method is utilized to combine the outputs in an appropriate manner for optimal results.

#### 4.4.3. Defuzzification

**Figure 15:**
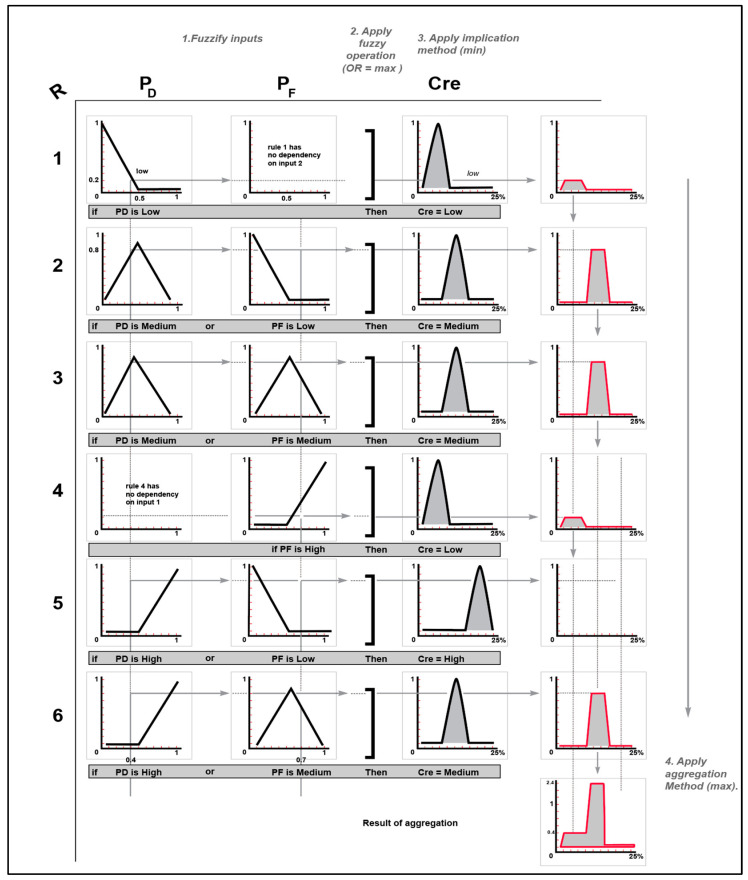
Illustration of fuzzy logic in event detection.

The process of defuzzification takes a fuzzy set as input, specifically the aggregate output fuzzy set, and generates a single numerical value as output. Although fuzziness is advantageous during the intermediate stages of rule assessment, a singular numerical value is typically the desired outcome for each variable. However, since an aggregate fuzzy set can have a broad range of output values, it must be DE fuzzified to obtain a single output value from the set. Once the set of rules has been designed, the output indicates the likelihood that an event detection will yield the following percentages: Low (56%), Medium (31%), and High (13%). To obtain a single, precise value, a defuzzification process is employed at this stage. Defuzzification techniques such as center of gravity, center of singleton, and maximal methods are commonly used. For this study, the defuzzification process was inspired by [1] and is used to obtain the credibility (*Cre*) of the analyzed cluster k. The process of using the complete set of rules for a particular case with assumed inputs of WDP = 0.4 and WFP = 0.7 is illustrated in Figure 15. This example demonstrates how fuzzy logic works and outlines the steps for determining the credibility and output result.

### 4.5. Final Decision

The final decision is determined based on the output of each cluster, represented by *Cre_j_*, and the corresponding weight values, denoted as *W_j_*. A cluster with a higher credibility holds more weight in the decision-making process, while clusters with a lower credibility contribute less to the final decision computation. When a cluster is too far from the event source to detect it and its credibility is poor, it is given less weight. In this scenario, only the clusters with High and Medium levels of credibility are considered for the final decision. Suppose there are NC clusters, and m represents the cluster associated with a low credibility. In that case, the weight of the *j-th* cluster (*w_j_*) is computed by normalizing the credibility as mentioned in Equation (3e).

The final decision is determined based on the readability of each cluster (*j-th* cluster), represented by *Cre_j_*, and the maximum credibility among m clusters, represented by *Cre_max_*. The following simple weighted OR rule is used to make the final decision:

(4B) $$H=\left\{\begin{array}{c} {\;\;H}_{1}\;\;\;\;\;\;\;\;\;\;\;\;\;\;\;\;\;\;\;\;\mathrm{if}\;\sum_{j=1}^{k}w_{j}D_{j}\geq 1\\ H_{0}\;\;\;\;\;\;\;\;\;\;\;\;\;\;\;\;\;\;\;\;\;\;\;\;\;\;\;\;\;\;\;\;\mathrm{otherwise} \end{array}\right.$$

### 4.6. Design of Event Detection for Fire: A Case Study

This section describes a case study of fire detection for indoor scenarios and considers a uniformly deployed sensor network to monitor the targeted area and trigger an alarm if a fire starts. In fact, fire can be defined as a sequence of exothermic chemical reactions between fuel and an oxidant accompanied by the by-products of combustion, which may result heat, smoke, and electromagnetic radiation (light) [9] in addition to gas. Therefore, different types of sensors could be used in fire detection to sense fire events such as temperature, smoke, light, and gas sensors. 

### 4.7. Fire Detection Procedures and Numerical Example

According to the proposed event detection structure, the detection mechanism consists of three major parts: (i) sensor-level detection; (ii) cluster head detection; and (iii) fusion center. For sensor-level detection, four types of sensors (smoke (S), temperature (T), light (L), and gas (G)) were deployed in each sub-region. The following procedures were used in the case study according to the designed event detection mechanism.

Sensor nodes send their measured data to their corresponding cluster head. Thus, the detection probability *H*_1_ and false alarm probability *H*_0_ information at the cluster head (after solving the *Q* Function and its equivalent error function as a complementary CDF using Equations (4j) and (4m)) can be presented as follows:

(4C) $$H_{0}^{k}=\left\{\begin{array}{l} H_{0}^{S}\;:\;P_{F}^{i,\;S}\left(e_{i}^{S}\geq \lambda_{S\;}\right)=Q\left(\frac{\lambda_{S}-\mu }{\sigma }\right)=\frac{1}{2}\;\left(1-erf\left(\frac{\lambda_{S}-\mu }{\sqrt{2}\;.\sigma }\right)\right)\;\\ H_{0}^{T}\;:\;P_{F}^{i,\;T}\left(e_{i}^{T}\geq \lambda_{T\;}\right)=Q\left(\frac{\lambda_{T}-\mu }{\sigma }\right)=\frac{1}{2}\;\left(1-erf\left(\frac{\lambda_{T}-\mu }{\sqrt{2}\;.\sigma }\right)\right)\\ H_{0}^{L}\;:\;P_{F}^{i,\;L}\left(e_{i}^{L}\geq \lambda_{L\;}\right)=Q\left(\frac{\lambda_{L}-\mu }{\sigma }\right)=\frac{1}{2}\;\left(1-erf\left(\frac{\lambda_{L}-\mu }{\sqrt{2}\;.\sigma }\right)\right)\\ H_{0}^{O}\;:\;P_{F}^{i,\;O}\left(e_{i}^{O}\geq \lambda_{O\;}\right)=Q\left(\frac{\lambda_{O}-\mu }{\sigma }\right)=\frac{1}{2}\;\left(1-erf\left(\frac{\lambda_{O}-\mu }{\sqrt{2}\;.\sigma }\right)\right) \end{array}\right.$$

(4D) $$H_{1}^{k}=\left\{\begin{array}{l} H_{1}^{S}\;:\;P_{F}^{i,\;S}\left(e_{i}^{S}\geq \lambda_{S\;}\right)=1-Q\left(\frac{\lambda_{S}-\mu -{e\;}_{i}^{S}\left(d\right)\;}{\sigma }\right)=\frac{1}{2}\;\left(1-erf\left(\frac{\lambda_{S}-\mu -{e\;}_{i}^{S}\left(d\right)}{\sqrt{2}\;.\sigma }\right)\right)\;\\ H_{1}^{T}\;:\;P_{F}^{i,\;T}\left(e_{i}^{T}\geq \lambda_{T\;}\right)=1-Q\left(\frac{\lambda_{T}-\mu -{e\;}_{i}^{T}\left(d\right)\;}{\sigma }\right)=\frac{1}{2}\;\left(1-erf\left(\frac{\lambda_{T}-\mu -{e\;}_{i}^{T}\left(d\right)\;}{\sqrt{2}\;.\sigma }\right)\right)\\ H_{1}^{L}\;:\;P_{F}^{i,\;L}\left(e_{i}^{L}\geq \lambda_{L\;}\right)=1-Q\left(\frac{\lambda_{L}-\mu -{e\;}_{i}^{L}\left(d\right)\;}{\sigma }\right)=\frac{1}{2}\;\left(1-erf\left(\frac{\lambda_{L}-\mu {-e\;}_{i}^{L}\left(d\right)}{\sqrt{2}\;.\sigma }\right)\right)\\ H_{1}^{O}\;:\;P_{F}^{i,\;O}\left(e_{i}^{O}\geq \lambda_{O\;}\right)=1-Q\left(\frac{\lambda_{O}-\mu -{e\;}_{i}^{O}\left(d\right)\;}{\sigma }\right)=\frac{1}{2}\;\left(1-erf\left(\frac{\lambda_{O}-\mu {-e\;}_{i}^{O}\left(d\right)}{\sqrt{2}\;.\sigma }\right)\right) \end{array}\right.$$

Table 3 illustrates the mean values and variances of different types of sensors according to data presented in the NIST dataset [26].

**Figure 16:**
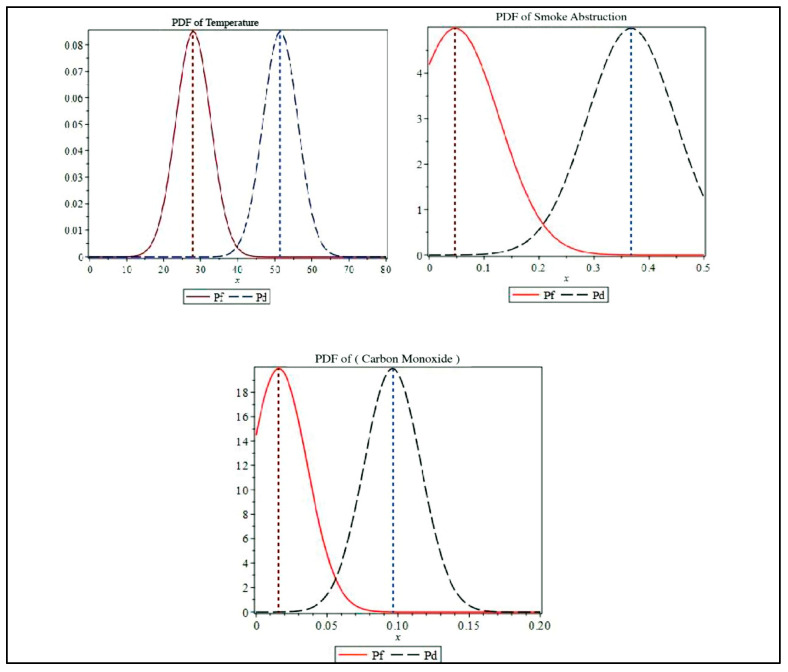
PDF of detection probability and false alarm probability for temperature, smoke, and gas.

The PDFs of the three sensor values (shown in Figure 16), which were obtained from the Test 5 datasheet from NIST, illustrate the designed PDF for detection probability and false alarm probability.

For instance, two reading values (one before the fire event and the other after the ignition of the fire) can be used to understand the behavior of the designed model. For this example, Maple software was used to solve the equations and obtain the following results.

Case 1 (no fire): Temperature (22.4 °C), smoke obstruction (0.0205%/m), and CO (0.0151). By applying Equations (4B) and (4C): 

Temperature: (*H*_0_ = 0.703456810, *H*_1_ = 6.140 × 10^−7^); 

Smoke: (*H*_0_ = 0.8026330517, *H*_1_ = 6.784 × 10^−7^); 

Carbon Monoxide (CO): (*H*_0_= 0.5179463455, *H*_1_ = 0.261616 × 10^−4^).

Case 2 (fire): Temperature (51.9 °C), smoke obstruction (0.482%/m), CO (0.0235).

Temperature: (*H*_0_ = 1.837 × 10^−7^, *H*_1_ = 0.8610627296);

Smoke: (*H*_0_ = 2.70 × 10^−8^, *H*_1_ = 0.9247120136); 

Carbon Monoxide (CO): (*H*_0_ = 0.1444808 × 10^3^, *H*_1_ = 0.3538302334).

At the second phase, a decision is made at the *CH* such that a positive detection of an event is signaled as soon as enough sensors have a high detection probability; at this phase, the decision mainly depends on high detection values that are received from the majority of local sensors. Accordingly, in the proposed model, every sensor type will send P_D_ and P_F_ values to its corresponding *CH*. Hence, the *CH* converts these values into linguistic terms (Low or High) based on Equation (4n). Then, the decision at the *CH* is positive if two out of three sensors have a detection probability that is High, for instance, temperature and smoke or CO and smoke. Thus, the decision will be as follows:

Case 1: All three sensors are determined to have a High false alarm and a Low detection probability, so the decision at the *CH* is *H*_0_ (no fire). 

Case 2: Two sensors (temperature and smoke) have High detection, so the decision at the *CH* is *H*_1_ (fire).

- Every cluster consists of three different types of sensors; the average detection probability *Q*_1_ and average false alarm probability *Q*_0_ can be computed for cluster k, using Equations (4p) and (4q) as follows:

Case 1:

$$\begin{array}{c} Q_{0}=\frac{H_{0}^{S}+H_{0}^{T}+H_{0}^{CO}}{3}=0.650\\ Q_{1}=\frac{H_{1}^{S}+H_{1}^{T}+H_{1}^{CO}}{3}=0.000011 \end{array}$$

Case 2:

$$\begin{array}{c} Q_{0}=\frac{H_{0}^{S}+H_{0}^{T}+H_{0}^{CO}}{3}=0.118\\ Q_{1}=\frac{H_{1}^{S}+H_{1}^{T}+H_{1}^{CO}}{3}=0.595 \end{array}$$

Fusion Centre: the level is applied in the fusion center as described below.

- At this stage, the WDP and WFP are computed by applying Equations (4z) and (4A); for this numerical example, only one cluster is considered, so W is equal 1 and therefore, WDF = *Q*_1_ and WFP = *Q*_0_.
- WDF = *Q*_1_ and WFP = *Q*_0_ were used as inputs to the fuzzy logic to determine the final decision for the cluster.

**Figure 17:**
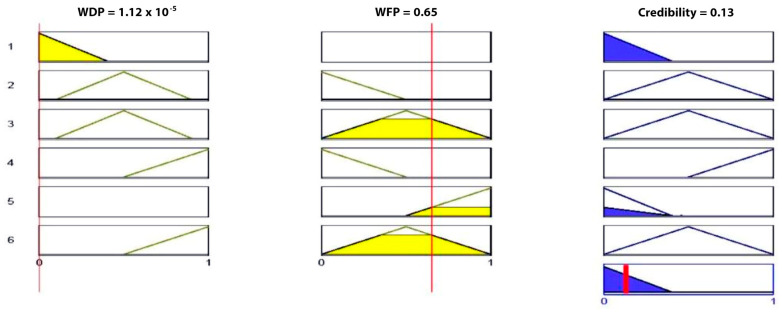
Implementation of fuzzy rule for Case 1.

**Case 1:** After applying the fuzzy process and the processes of fuzzification and defuzzification (Figure 17), the output was equal to 0.13, which indicates that there is no fire.

**Figure 18:**
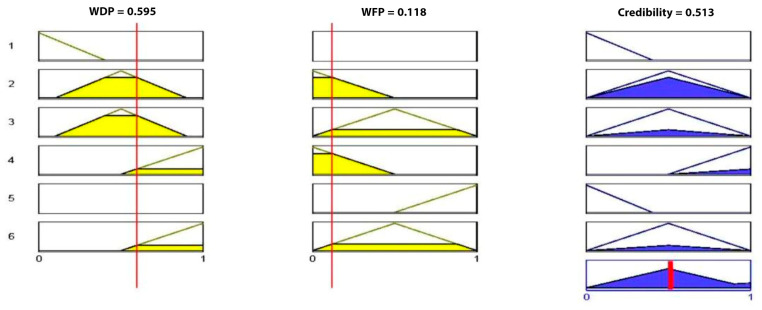
Implementation of fuzzy rule for Case 2.

**Case 2**: In this case, the output was equal to 0.513, which indicates the presence of fire (Figure 18).

In brief, this numerical example illustrates the operation of the proposed models for event detection and validates that the models and designed mathematical models are working properly in the cases of absence of fire and presence of fire.

## 5. Analysis and Findings from Experimental Study

This study’s proposed work consists of two phases: firstly, gathering the event environment dataset, and secondly, implementing the proposed design using MATLAB software. To illustrate the effectiveness of the proposed approach, a case study of fire event detection was conducted.

### 5.1. Dataset

The NIST website (“Home Smoke Alarm Tests|NIST”, Ref. [26]) provides a real fire dataset that was obtained through sensor measurements from various real fires. This study focused on a factory building and selected two primary scenarios from the available options, namely, fire caused by a burning mattress and fire caused by a burning chair.

### 5.2. Experiment

A simulated home was constructed with a variety of heterogeneous sensors deployed in two locations. The geometry of the simulated home was representative of a range of residential layouts, including apartments. The home was partitioned into one living room, three bedrooms (each bedroom also containing a small closet), one full bathroom, one kitchen/dining area, and two hallways. The collected data were divided based on the most frequent fires and those that resulted in the highest number of fatalities. It was found that fires involving flaming and smoldering upholstered furniture and mattresses were among the top four deadliest fire scenarios [27]. In line with [28], the simulation time for all experiments was set at 600 s. The parameter settings for the evaluation experiments are summarized in Table 4.

### 5.3. Metrics for Evaluation

In order to assess the PCED mechanism’s reliability, efficiency, and performance, three metrics (or criteria) were utilized to analyze the simulation results:

- Accuracy

“Accuracy” is a widely used measure of how closely a measurement aligns with the true value. In the context of the proposed PCED mechanism, accuracy was calculated by assessing the number of correctly detected events, based on detection probability, number of sensors, and clustering. As explained in reference [29,30], the general formula for calculating accuracy is as follows:

(5) $$Accuracy=\frac{Total\;Number\;of\;True\;Alarms}{Total\;Number\;of\;Event\;Detection}*100\%$$

- Detection Delay

The delay of an event is the duration between the occurrence of a physical event and its detection [31]. In the context of fire monitoring, timely reporting to the fusion center is crucial. Hence, one of the primary performance metrics for monitoring event-based Wireless Sensor Networks (WSNs) is the detection delay [32]. This metric measures the time elapsed between the physical occurrence of an event and the delivery of a sufficient number of packets to the fusion center.

- False Alarms

An event behavior that is mistakenly classified as abnormal by the fusion center can occur due to inaccurate data from sensor nodes and the potential evolution of the event relative to the number of detecting nodes [33]. The fusion center depends on the accuracy of the data collected from the sensor nodes to produce the classification decision [34], which may be affected by the arrangement of the nodes detecting the event and their function.

### 5.4. Results and Performance Evaluation

In this section, we compared the performance of the proposed event detection method, PCED, and an existing method named REDF. The comparison was based on the detection output from fuzzy logic, the detection delay, and false alarm rate. The NIST dataset was utilized to simulate the monitoring environment of a home. To ensure a fair comparison, only one cluster was considered, following the scenario of REDF. REDF was implemented in MATLAB using fuzzy logic, as described in [14].

#### 5.4.1. Probability of Final Decision Detection 

**Figure 19:**
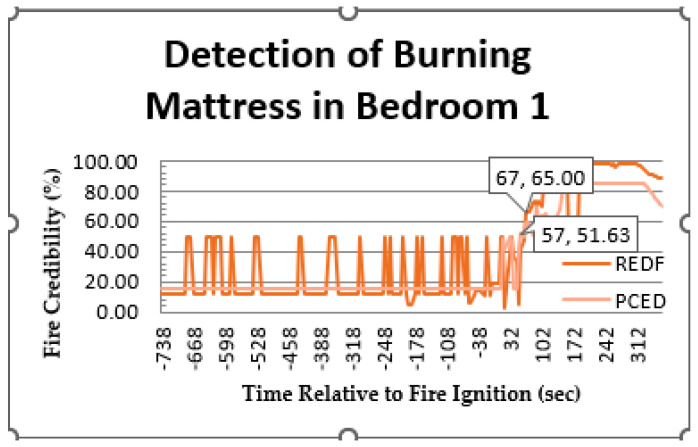
Comparison of fire detection performance for PCED and REDF methods: credibility values for burning mattress.

**Figure 20:**
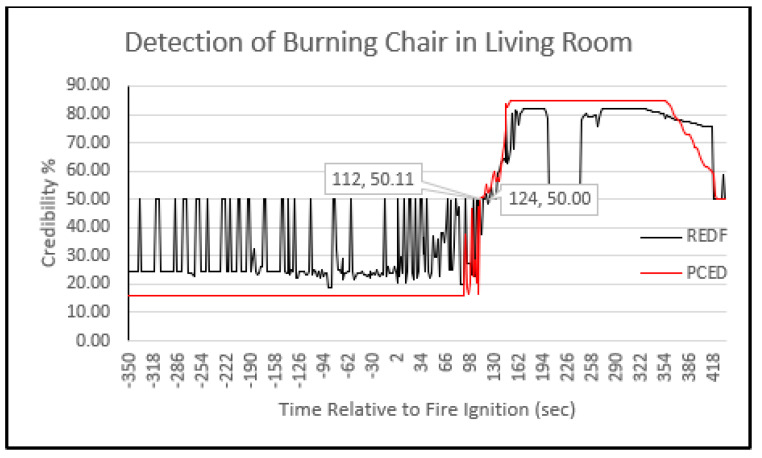
Evaluating the credibility of a burning chair’s PCED and REDF values.

In Figure 19 and Figure 20, the fuzzy output results in the fusion center are presented as percentages. The *x*-axis represents the time in seconds relative to the ignition of a fire, while the *y*-axis shows the fire credibility as a percentage. Figure 19 shows the results for a burning mattress in bedroom 1, comparing the proposed PCED mechanism to REDF. Negative time values indicate the period before the fire ignition. In both mechanisms, a credibility threshold of 50% was used to trigger the detection of a fire. Credibility values above 50% indicate the presence of a fire, while values below 50% indicate the absence of fire.

The results shown in Figure 19 demonstrate the superiority of the proposed PCED mechanism over the existing REDF method in terms of system output stability during the absence of events, detection delay, and false alarms for events prior to fire ignition. The proposed PCED mechanism detected the presence of fire at 57 s, whereas REDF detected it at 67 s, indicating a 10 s delay. This performance improvement can be attributed to the proposed PCED model’s use of different types of sensor nodes, allowing it to gather more event information than REDF, which relies solely on temperature and requires more time to detect an increase in heat.

An important observation is that the credibility behavior in the proposed PCED method was more stable during the absence of fire and after the fire was detected. This stability can be attributed to the probability design at the sensor nodes, which ensures that the detection probability and false alarm values are determined in a way that minimizes their interference. This observation indicates that there was an increase in the credibility of PCED after fire ignition, but it did not reach the threshold value of 50% which is necessary for fire detection. Consequently, PCED did not consider it a fire. However, at certain points or sometime after the fire started, the credibility of PCED decreased to a lower level of around 15%, which is referred to as a negative alarm. Comparatively, REDF experienced eight negative alarms, while PCED encountered only two. These negative alarms were triggered by unexpected changes in sensor readings. The output result of the burning chair credibility in the living room is depicted in Figure 20. It is evident from the results that the detection delay in this case was higher than the previous case presented in Figure 10, with values of 112 s and 124 s for PCED and REDF, respectively. The delay in detecting a fire can be assigned to many factors such as the kind of material being burnt, the area’s size, and the adjacent environmental situations, which may influence the sensor readings and result in fluctuations. Consequently, it is essential for the event detection mechanism to be resilient against these variables to improve the accuracy of the final decision. Although the PCED’s credibility score began to rise at the 92 s mark, it decreased multiple times, resulting in further delay in detecting the fire. 

Table 5 displays the detection delay for both the proposed PCED and REDF mechanisms, with the results indicating that the former detected the fire 10 s earlier for the mattress case and 12 s earlier for the burning chair case compared to REDF. This improvement resulted in a delay reduction of 17.5% for the burning mattress and 10.7% for the burning chair scenarios. Table 5 also presents the detection probability, which is calculated by dividing the detection time by the experiment time from the ignition of the fire. The proposed PCED method outperformed REDF in terms of detection probability as well.

#### 5.4.2. Detection of False Alarms

In Table 6, a summary of the false alarms detected prior to fire ignition is presented and indicates that REDF exhibited a higher false alarm rate than PCED. Possible reasons for this disparity include issues related to the design of the detection mechanism and the selection of sensing value ranges. One of the key parameters for fire detection is the change in values such as smoke levels. For instance, a smoke value that is initially close to zero but then changes significantly can indicate the presence of fire. Additionally, a probabilistic approach can help the system manage the sensing data by converting the values into probabilities that determine the detection probability and false alarm rates for all values. The selection of probability parameters and probability design, particularly mean values, can significantly improve the accuracy of fire detection mechanisms in distinguishing between detection and false alarm probabilities. However, the design of REDF may encounter issues with fuzzy logic membership functions that incorporate negative values, resulting in erroneous output credibility values. The high false alarm rate associated with REDF is due to the fact that it often produced false positives for zero smoke and temperature values that experience negative changes. Consequently, REDF yielded values that are close to 50% just prior to fire ignition, thereby elevating the false alarm rate. Conversely, the proposed PCED maintained low credibility values when no fire detection occurred and increased them to the highest level once a fire was detected. Moreover, PCED incorporates a weighting factor that assigns higher detection probabilities to clusters that detect events and lower probabilities to clusters that do not. As demonstrated in Table 5, PCED significantly reduced false alarms from 37 to 3 instances in the case of the burning mattress and eliminated them altogether in the case of the burning chair.

Based on the results presented above, we can calculate the detection accuracy by dividing the total number of true detections, including both fire and no fire, by the sum of all types of detections, including true detections, false alarms, and negative alarms. Table 7 compares the detection accuracy of the proposed PCED with the related REDF based on the NIST dataset results. In the scenario of the burning mattress, PCED demonstrated superior accuracy, with an accuracy of 94.4%, while REDF achieved only 79%. REDF’s inferior performance was due to its high rates of false alarms and negative alarms. However, for the case of the burning chair, PCED’s accuracy was slightly lower, which can be attributed to detection delay. Overall, the performance of PCED surpassed that of REDF in both scenarios. The accuracy enhancement achieved by PCED over REDF was 19.4% for the burning mattress case and 8.1% for the burning chair case. These results indicate that the proposed PCED was superior to REDF in terms of performance.

## 6. Conclusions

The objective of this study was to propose a probabilistic collaborative event detection mechanism (PCED) that can efficiently and effectively reveal events in indoor environments using WSNs while decreasing false event probability and detection delay. This study focused on achieving high levels of performance and accuracy in event detection. The proposed PCED mechanism involves three levels: local detection, cluster decision, and final fusion decision. The obtained results from several scenarios were analyzed and discussed. The performance of the PCED mechanism was assessed based on detection accuracy, event detection delay, and false alarms in whole experimental evaluations. The evaluations help to prove the PCED mechanism’s effective execution in challenging scenarios of event detection using WSNs. The results revealed that the PCED mechanism performs well in terms of detection accuracy, with an increase in detection probability and a decrease in detection delay and false alarms. It is worth noting that event detection systems in practical applications should typically generate alarms or notifications as part of their outputs. In this study, the PCED mechanism accurately detects events, but the notification method and type of reactions are not specified at the application level. This is because it depends on the specific application’s purpose, deployment, and system applicability.

As a result, it is worthwhile to investigate appropriate methods for responding to events as they occur. This could involve developing an effective communication system that alerts the emergency team with sufficient information or implementing an intelligent system capable of mitigating the impact of combustion gases, regulating oxygen levels around the fire event, and preventing the transfer of glowing heat.

## Figures and Tables

**Table 1:** Rule set for fuzzy inference.

Rule	*WDP*	*WFP*	*Cre*
1	L	L	L
2	L	M	L
3	L	H	L
4	M	L	M
5	M	M	M
6	M	H	L
7	H	L	H
8	H	M	M
9	H	H	L

**Table 2:** Fuzzy inference rule set with exclusion of rules 1, 3, and 6.

Rule	*WDP*	*WFP*	*Cre*
1	L	-	L
2	M	L	M
3	M	M	M
4	-	H	L
5	H	L	H
6	H	M	M

**Table 3:** Statistical values for different types of sensors.

Sensing Type	Mean Value (µ)	Standard Deviation (σ)	Threshold Value
Temperature	28 °C	4.7 °C	55 °C
Smoke	0.0474/m	0.0822/m	0.15/m
Gas (Carbon Monoxide)	0.016	0.0245	-

**Table 4:** Distribution of cluster heads in monitoring area.

Parameters	Values
	Simulated Home
Simulation Time (s)	600
Location of fire	Bedroom, Living Room
Sensor types	Optical Density, Smoke, Temperature, Gas
Topology Network	Cluster-based
Distribution types	Gaussian Normal Distribution
Heat Release Rate Per Unit Area (HRRPUA) (kw/m^2^)	745.25

**Table 5:** Summary of detection delay results for manufactured homes using NIST dataset.

Burning Material	Detection Delay (Seconds)		Detection Probability	
	PCED	REDF	PCED	REDF
Mattress (Bedroom 1)	57	67	87.8%	84.7%
Chair (Living Room)	112	124	74.5%	71.8%

**Table 6:** False alarm summary for manufactured homes using NIST dataset.

Burning Material	False Alarm	
	PCED	REDF
Mattress (Bedroom 1)	03	37
Chair (Living Room)	0	45

**Table 7:** Assessing detection accuracy in a manufactured home using NIST dataset.

Burning Material	Accuracy of Detection	
	PCED	REDF
Mattress (Bedroom 1)	94.4%	79%
Chair (Living Room)	93%	86%

## References

[1] Thuc K.-X., Insoo K. (2011). A collaborative event detection scheme using fuzzy logic in clustered wireless sensor networks. AEU-Int. J. Electron. Commun..

[2] Prabhu B. (2016). Research Insights in Clustering for Sparsely Distributed Wireless Sensor Network. Int. J. Adv. Eng. Res..

[3] Prabhu B., Balakumar N. (2016). Highly Scalable Energy Efficient Clustering Methodology for Sensor Networks. Int. J. Adv. Eng. Res..

[4] Wu H., Cao J., Fan X. (2016). Dynamic collaborative in-network event detection in wireless sensor networks. Telecommun. Syst..

[5] Polastre J., Szewczyk R., Culler D. Telos: Enabling ultra-low power wireless research. Proceedings of the IPSN 2005 Fourth International Symposium on Information Processing in Sensor Networks.

[6] Bernardo L., Oliveira R., Tiago R., Pinto P. (2007). A fire monitoring application for scattered wireless sensor networks-a peer-to-peer cross-layering approach. Int. Conf. Wirel. Inf. Netw. Syst..

[7] Wittenburg G., Dziengel N., Wartenburger C., Schiller J. A system for distributed event detection in wireless sensor networks. Proceedings of the 9th ACM/IEEE International Conference on Information Processing in Sensor Networks.

[8] Li S., Son S.H., Stankovic J.A. (2003). Event detection services using data service middleware in distributed sensor networks. Information Processing in Sensor Networks.

[9] Fristedt B., Gray L. (1997). Renewal Sequences. A Modern Approach to Probability Theory.

[10] Guo J., Jafarkhani H. (2016). Sensor deployment with limited communication range in homogeneous and heterogeneous wireless sensor networks. IEEE Trans. Wirel. Commun..

[11] Wang T., Peng Z., Wang C., Cai Y., Chen Y., Tian H., Liang J., Zhong B. (2016). Extracting Target Detection Knowledge Based on Spatiotemporal Information in Wireless Sensor Networks. Int. J. Distrib. Sens. Netw..

[12] Cheng S., Cai Z., Li J., Fang X. Drawing dominant dataset from big sensory data in wireless sensor networks. Proceedings of the 2015 IEEE Conference on Computer Communications (INFOCOM).

[13] Marin-Perianu M., Havinga P. (2007). D-FLER—A distributed fuzzy logic engine for rule-based wireless sensor networks. Ubiquitous Comput. Syst..

[14] Kapitanova K., Son S.H., Kang K.-D. (2012). Using fuzzy logic for robust event detection in wireless sensor networks. Ad. Hoc. Networks.

[15] Brass P. (2007). Bounds on coverage and target detection capabilities for models of networks of mobile sensors. ACM Trans. Sens. Networks.

[16] Liu M., Cao J., Lou W., Chen L.J., Li X. (2005). Coverage analysis for wireless sensor networks. Proceedings of the Mobile Ad-hoc and Sensor Networks: First International Conference, MSN 2005.

[17] Lazos L., Poovendran R., Ritcey J.A. Probabilistic detection of mobile targets in heterogeneous sensor networks. Proceedings of the 6th International Conference on Information Processing in Sensor Networks.

[18] Varshney P.K. (2007). Distributed Detection and Data Fusion.

[19] Yi S., Hao Z., Qin Z., Li Q. Fog computing: Platform and applications. Proceedings of the Third IEEE Workshop on Hot Topics in Web Systems and Technologies (HotWeb).

[20] Yuan Z., Tan R., Xing G., Lu C., Chen Y., Wang J. Fast sensor placement algorithms for fusion-based target detection. Proceedings of the 2008 Real-Time Systems Symposium.

[21] Tan W., Wang Q., Huang H., Guo Y., Zhang G. Mine fire detection system based on wireless sensor network. Proceedings of the 2007 International Conference on Information Acquisition.

[22] Yang G., Qiao D. Barrier information coverage with wireless sensors. Proceedings of the IEEE INFOCOM 2009.

[23] Dampage U., Bandaranayake L., Wanasinghe R., Kottahachchi K., Jayasanka B. (2022). Forest fire detection system using wireless sensor networks and machine learning. Sci. Rep..

[24] Zervas E., Sekkas O., Hadjieftymiades S., Anagnostopoulos C. Fire detection in the urban rural interface through fusion techniques. Proceedings of the 2007 IEEE International Conference on Mobile Adhoc and Sensor Systems.

[25] Fishman G.S. (2013). Discrete-Event Simulation: Modeling, Programming, and Analysis.

[26] Home Smoke Alarm Tests. https://www.nist.gov/engineering-laboratory/home-smoke-alarm-tests.

[27] Bukowski R., Peacock R., Averill J., Cleary T., Bryner N., Walton W., Reneke P., Kuligowski E. (2007). Performance of Home Smoke Alarms Analysis of the Response of Several Available Technologies in Residential Fire Settings (NIST TN 1455-1), Technical Note (NIST TN).

[28] Mcgrattan K., Hostikka S., Floyd J., Baum H., Rehm R. (2007). Fire Dynamics Simulator (Version 5) Technical Reference Guide.

[29] Singh V.K., Kumar M., Verma S. (2017). Accurate Detection of Important Events in WSNs. IEEE Syst. J..

[30] Gungor V.C., Akan Ö.B., Akyildiz I.F. (2008). A Real-Time and Reliable Transport (RT) 2 Protocol for Wireless Sensor and Actor Networks. IEEE/ACM Trans. Netw..

[31] Al Samara M., Bennis I., Abouaissa A., Lorenz P. (2023). Complete outlier detection and classification framework for WSNs based on OPTICS. J. Netw. Comput. Appl..

[32] Debasis K., Sharma L.D., Bohat V., Bhadoria R.S. (2023). An Energy-Efficient Clustering Algorithm for Maximizing Lifetime of Wireless Sensor Networks using Machine Learning. Mob. Netw. Appl..

[33] Balakumar N., Boselin Prabhu S. (2016). Literature and Comparative Survey of Future Wireless Communication. Galaxy Int. Multidiscip. Res. J..

[34] Elavarasan S., Balakumar N. (2016). A Research on Wireless Power Transmission using Distinguished Methodologies. Int. J. Res. Eng..

